# Visuospatial Working Memory in Toddlers with a History of Periventricular Leukomalacia: An EEG Narrow-Band Power Analysis

**DOI:** 10.1371/journal.pone.0069837

**Published:** 2013-07-26

**Authors:** María Luisa García-Gomar, Efraín Santiago-Rodríguez, Mario Rodríguez-Camacho, Thalía Harmony

**Affiliations:** 1 Unidad de Investigación en Neurodesarrollo “Dr. Augusto Fernández Guardiola”, Departamento de Neurobiología Conductual y Cognitiva, Instituto de Neurobiología, Universidad Nacional Autónoma de México (UNAM), Querétaro, México; 2 Facultad de Estudios Superiores Iztacala, Universidad Nacional Autónoma de México (UNAM), Estado de México, México; Cuban Neuroscience Center, Cuba

## Abstract

**Background:**

Periventricular Leukomalacia (PVL) affects white matter, but grey matter injuries have also been reported, particularly in the dorsomedial nucleus and the cortex. Both structures have been related to working memory (WM) processes. The aim of this study was to compare behavioral performances and EEG power spectra during a visuospatial working memory task (VSWMT) of toddlers with a history of PVL and healthy toddlers.

**Methodology/Principal Findings:**

A prospective, comparative study of WM was conducted in toddlers with a history of PVL and healthy toddlers. The task responses and the EEG narrow-band power spectra during a VSWMT were compared in both groups. The EEG absolute power was analyzed during the following three conditions: baseline, attention and WM retention. The number of correct responses was higher in the healthy group (20.5±5.0) compared to the PVL group (16.1±3.9) (p = 0.04). The healthy group had absolute power EEG increases (p≤0.05) during WM compared to the attention condition in the bilateral frontal and right temporal, parietal and occipital regions in frequencies ranging from 1.17 to 2.34 Hz and in the right temporal, parietal and occipital regions in frequencies ranging from 14.06 to 15.23 Hz. In contrast, the PVL group had absolute power increases (p≤0.05) in the bilateral fronto-parietal, left central and occipital regions in frequencies that ranged from 1.17 to 3.52 Hz and in the bilateral frontal and right temporal regions in frequencies ranging from 9.37 to 19.14 Hz.

**Conclusions/Significance:**

This study provides evidence that PVL toddlers have visuospatial WM deficits and a very different pattern of absolute power increases compared to a healthy group of toddlers, with greater absolute power in the low frequency range and widespread neuronal networks in the WM retention phase.

## Introduction

One of the most common brain injuries in premature infants is periventricular leukomalacia (PVL), although it can also occur in full-term infants [1], [2]. PVL occurs in 50% of very low birth weight (VLBW) infants and is defined as necrosis of the white matter, although it has also been observed in the grey matter [3]. In addition to the white matter damage, PVL affects the cerebral cortex and the mediodorsal and reticular nuclei in the thalamus [4], [5]; these structures are involved in neuronal networks of cognitive processes, such as working memory (WM) and attention [6]. It has been reported that children with a history of PVL have deficits in object recognition, visual imagery and visuospatial memory [7].

WM is defined as the active retention of information for a prospective action, a problem solution or the achievement of a goal [8]. It starts to develop from five to six months of age, when infants are capable of reaching one object hidden in one of two possible locations after a delay from one to two seconds [9]. This task was named the delayed response task (DRT) [10], and this task, along with the Piagetian task A not B [11], is used to study WM. In this view, WM capacity can be defined as the tolerable delay, which is the amount of time in which the participant is capable of actively maintaining the information in his/her memory before correctly reaching for the hidden object. In infants, a linear improvement has been shown for the tolerable delay at a rate of two seconds per month from approximately two seconds at seven 1/2 months to 10 seconds at 12 months [12]. It has also been proposed that there are no significant changes in the execution from 5.5 to 8 months of age but that a linear increase occurs in the correct percentage of DRT trials from 8 to 12 months [13].

Electroencephalogram (EEG) is a technique to study brain activity in a non-invasive manner. Quantitative EEG analysis provides objective information about background EEG activity [14]. In infancy, during the execution of WM tasks, eight-month-old healthy infants have shown a generalized increase in the absolute power in the 6–9 Hz band in almost all cerebral regions [15]. In healthy two-year-old toddlers, execution of a verbal recall task has been related to 3–5 Hz and 6–9 Hz power increases [16]. Finally, in early childhood during the execution of inhibitory control tasks, 4.5-year-old healthy children have shown a power increase in the 6–9 Hz band, particularly in the fronto-medial leads, in addition to a coherence increase between the fronto-medial and posterior regions [17], [18]. All of these cerebral regions have been related to WM processes [8].

In summary, cognitive, sensory and motor problems have been reported in toddlers with a history of PVL as a result of white and grey matter damage. The aim of our study was to compare the behavioral performance and the EEG power spectra during a visuospatial working memory task (VSWMT) of toddlers with a history of PVL and healthy toddlers.

## Methods

### Subjects

Nineteen toddlers who attended the Unidad de Investigación en Neurodesarrollo “Dr. Augusto Fernández Guardiola”, Instituto de Neurobiología, Universidad Nacional Autónoma de México were included in the study. They were divided into the following two groups: 10 toddlers with a history of PVL with a mean age of 26±1.1 months and nine healthy toddlers with a mean age of 26.5±0.7 months. All children underwent a mental/motor development assessment with the Bayley Scales of Infant Development (BSID-II) [19], neurological and ophthalmologic examination with visual evoked potential study (VEPs) using pattern reversal and light-emitting diodes (LEDs) stimulation. To be classified into the PVL group, the toddlers had to have had abnormal neurological examinations and T2-weighted MRI images in which diffuse excessive high signal intensity (DEHSI) of the cerebral white matter was identified. The PVL toddlers had received neurohabilitation therapy since their first or second month of age and up to 12 to 20 months, until they had reached their motor independence [20]. The parents of all of the toddlers provided written informed consent, and the study was approved by the Institutional Bioethics Committee, Comité de Bioética, Institute of Neurobiology, National Autonomous University of México.

### Visuospatial Working Memory Task

**Figure 1:**
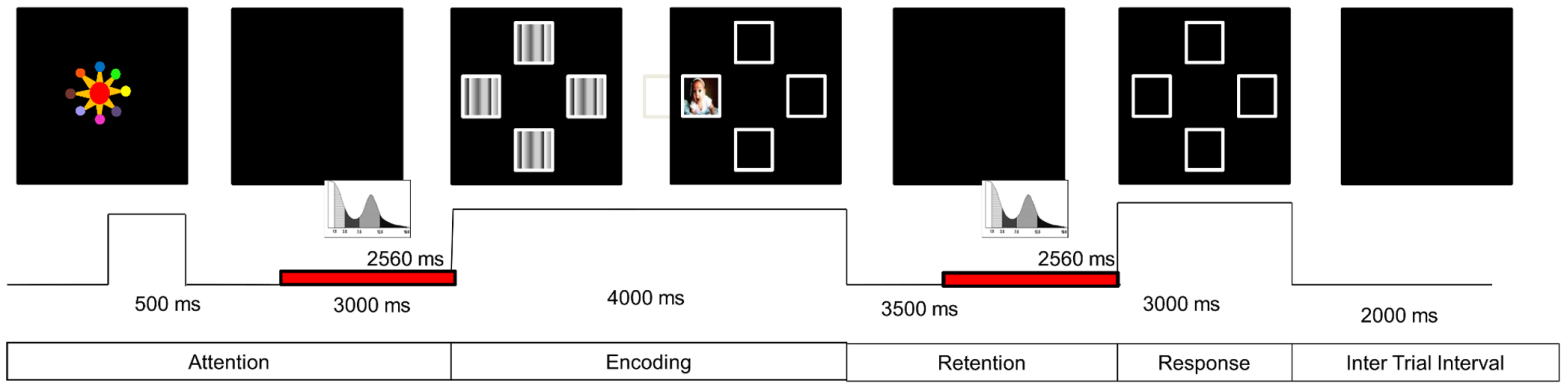
Visuospatial Working Memory Task (VSWMT). The durations and phases in which the windows were selected for the EEG power spectrum analysis in attention and retention (WM) conditions are shown in red. The salient stimulus of the encoding phase was taken from the IAPS [21].

The EEG synchronized task was performed in a sound-proof and darkened room. The toddlers sat on their parents’ legs at 41 cm in front of a 48-cm-wide color video monitor. The VSWMT was applied using Mind Tracer software (Neuronic Mexicana, SA, México City). The task included the following four main phases: attention, encoding, retention and response (Figure 1). In the attention phase, a colorful stimulus was presented for 500 ms in the center of the screen, and then the screen was kept black for 3000 ms. Then, four covered windows were presented for 2000 ms with 20 degrees of horizontal and vertical angular separation in the center top, bottom, left and right screen quadrants. In the encoding phase, a salient target stimulus was displayed in one of the four windows. In the retention phase, the screen was kept black for 3500 ms. Finally, in the response phase, the four uncovered windows appeared again for 3000 ms, and the toddlers gazed at the location where they thought the target stimulus had appeared in each trial. Correct responses were rewarded with verbal praises. The VSWMT showed the following seven salient target stimuli: images of puppies, kittens, babies, children and “Sesame Street” characters. Some of the images were taken from the International Affective Picture System (IAPS) [21]. The inter-trial interval was 2000 ms. The entire task consisted of 28 trials with a task duration of 7 min 32 s.

### Behavior Analysis

**Figure 2:**
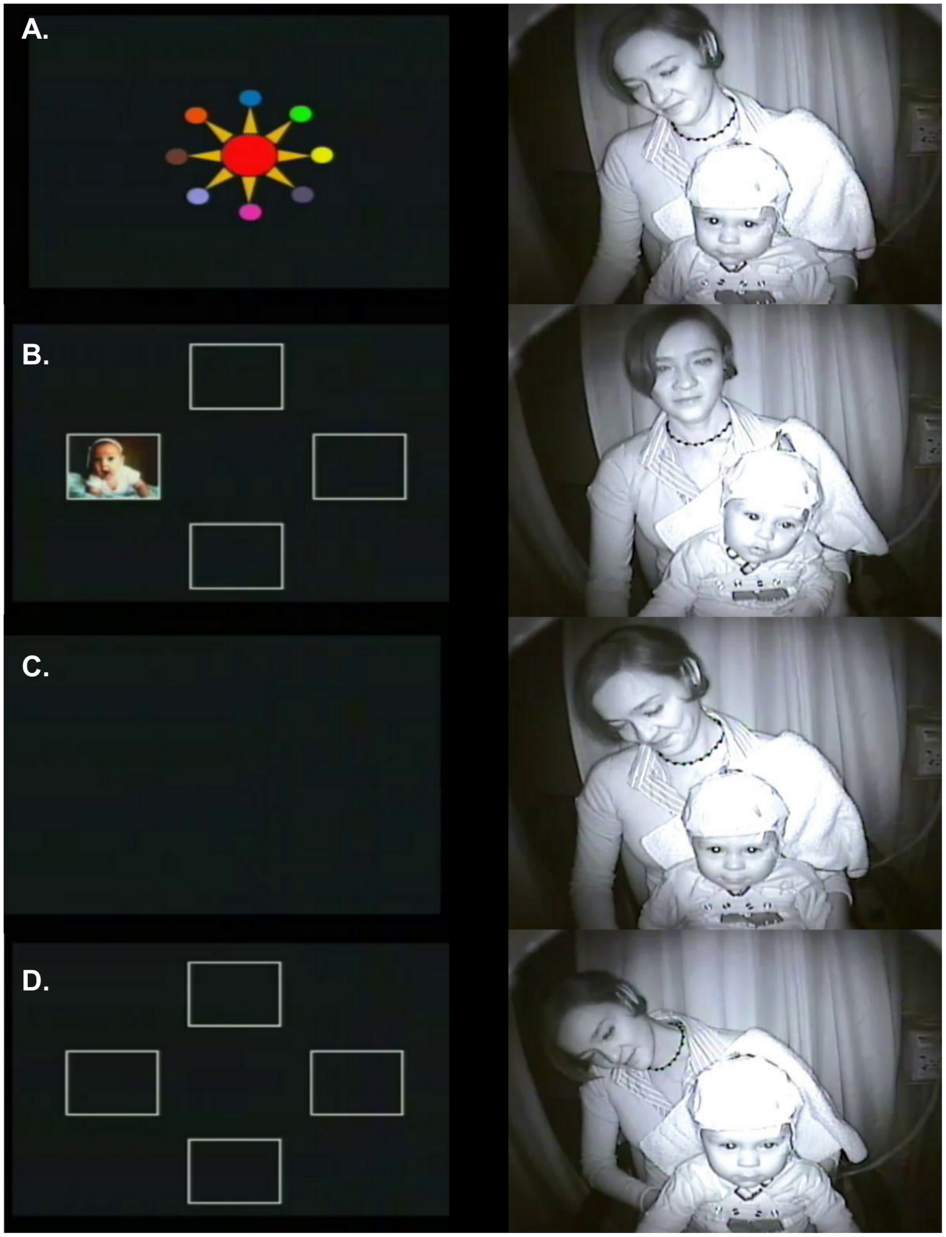
Correct answer in a healthy toddler. Left: Four phases of the VSWMT, as shown in the screen viewed by the toddlers. Right: Synchronized behavioral response of the toddler. A: attention phase, B: encoding phase, C: retention or WM retention phase and D: response phase. The subject of the photograph has given written informed consent, as outlined in the PLOS consent form, to publication of their photograph.

Gaze recording was accomplished using a hidden infrared video camera placed in front of the toddler. During the response phase, a correct response was defined as a gaze to the location where the target stimulus had appeared during the encoding phase in each trial (Figure 2). Types of incorrect responses were also analyzed and categorized into the following four types: no-encoding, perseverative error, non-perseverative error and no-response. No-encoding meant that the toddler did not encode the stimulus during the encoding phase. Perseverative errors meant that toddler properly encoded but, during the response phase, gazed at the location where the target had appeared in the immediately previous trial. Non-perseverative errors meant that toddler properly encoded the target, but during the response phase, gazed at a location where the target did not appear during the encoding phase in either the present trial or the immediately previous trial. Finally, no-responses meant that the toddler properly encoded but did not respond with a gaze and was crying or distracted.

### EEG Recording

The EEG data were collected using a stretch cap (Electro Cap International, Inc. Eaton, Ohio, USA) with 19 leads of the 10/20 international system, using linked earlobes as reference. Impedances were kept under 5 kΩ. The EEG data were recorded using the Medicid System (Neuronic Mexicana, S.A, México City), with a gain of 10,000 dB, low-cut filters at 0.5 Hz and high-cut filters at 30 Hz. The sample frequency was 200 Hz.

The EEG data were analyzed during the following three different conditions: baseline, attention and WM retention. Free artifact windows of 2560 ms were selected from the baseline EEG recording and during the attention and WM retention conditions of the VSWMT. Only correct-response trials were considered for analysis (Figure 1). The EEG power spectrum was obtained by a Fast Fourier Transformation of the 18 to 24 windows with a narrow-band power spectrum analysis between 0.781 and 19.14 Hz, at intervals of 0.390 Hz. The power was normalized by applying a geometric power to all of the leads [22]. The EEG data were analyzed using EP Workstation (Neuronic Mexicana, S.A, México City).

### Statistical Analysis

The inter-rater reliability of the correct responses was evaluated using the Cohen’s Kappa Coefficient. To assess differences between the groups for clinical characteristics of the sample and behavioral variables, the Mann-Whitney U test was used. The differences in the power spectra between the groups and between the baseline, attention and WM conditions were analyzed using Neest 2.0 (Neuronic Mexicana, México, City) for each of the 19 leads and for all of the narrow-band frequencies. A two-way ANOVA was calculated for the following two factors: group (PVL or healthy) and condition (baseline, attention or WM), and post-hoc analyses using t tests were conducted. This software displayed the results in topographic maps according to the significance level (p<0.05).

## Results

Toddlers in the PVL group had a lower gestational age (31±4.6 months) than did the healthy toddlers (38.2±1.3 months) (p = 0.002); they also had a lower birth weight (p = 0.0006) and lower Apgar scores at one (p = 0.001) and five minutes (p = 0.01) after birth. All toddlers had normal ophthalmologic evaluations and VEPs. The healthy toddlers had normal results in their neurologic examinations, and the PVL toddlers had lower scores (88.4±11.9) on the MDI (BSID-II Mental Developmental Index) than did the healthy toddlers (101.7±15.7) (p = 0.03). The clinical details of the toddlers are described in Table 1 and the MRI findings are shown in Table 2.

**Table 1:** Clinical characteristics of the toddlers.

	Healthy n = 9	PVL n = 10
Age (months)	26.5±0.7	26±1.1
Females	3	3
Gestational age (weeks)	38.2±1.3	31±4.6**
Birth weight (g)	3218.3±247.1	1689.5±776.5**
Apgar (1-min)	8.5±0.5	7±1.0**
Apgar (5-min)	9.1±0.3	8.3±0.9*
MDI	101.7±15.7	88.4±11.9*
PDI	105.3±8.9	98.7±11.0

* p≤0.05,

** p≤0.005. MDI: BSID-II Mental Developmental Index, PDI: BSID-II Psychomotor Developmental Index.

**Table 2:** Magnetic Resonance Image findings of toddlers with Periventricular Leukomalacia.

Subject	PCA	MRI Finding
1	38	DEHSI in white matter predominantly in left occipital region
2	42	DEHSI in white matter predominantly in right occipital region
3	38	DEHSI in white matter predominantly in right occipital region
4	48	Enlarged subarachnoid space, volume reduction of the corpus callosum
5	44	DEHSI in white matter, dilated lateral ventricles
6	42	DEHSI in white matter predominantly in occipital regions Enlarged subarachnoid space
7	37	DEHSI in white matter predominantly in right occipital region
8	42	DEHSI in white matter predominantly in occipital regions Enlarged subarachnoid space
9	38	DEHSI in white matter predominantly in occipital regions
10	42	DEHSI in white matter predominantly in occipital regions Enlarged subarachnoid space

PCA = Postconceptional age in weeks.

MRI = Magnetic Resonance Image.

DEHSI = Diffuse Excessive High Signal Intensity.

### Behavioral Performance

**Figure 3:**
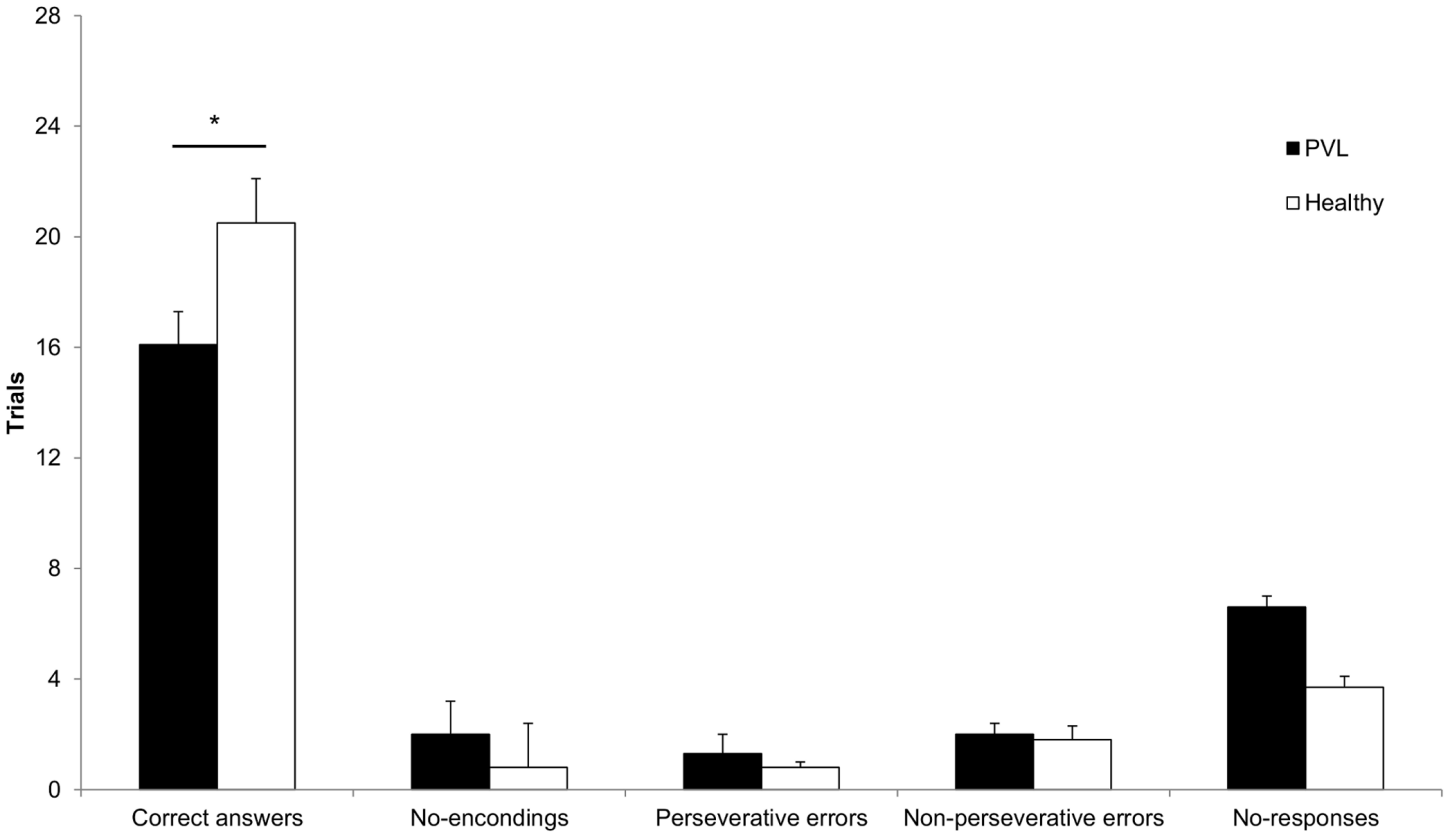
Correct and incorrect responses on the VSWMT. Responses to the VSWMT are shown in black for the PVL group and in white for the healthy group. The PVL group showed a lower number of correct responses than did the healthy group (*p≤0.05). For the different types of incorrect responses, no significant differences were identified.

The number of correct responses was lower in the PVL group (16.1±3.9; 57.5%) than in the healthy group (20.5±5.0; 73.2%) (p = 0.04). Regarding incorrect responses, the PVL group had similar numbers of no-encodings (p = 0.1), perseverative errors (p = 0.3), non-perseverative errors (p = 0.8) and no-responses (p = 0.06) compared to the healthy group (Figure 3). In addition, the number of trials required to complete the task was higher in the PVL group (44.1±9.2) compared to the healthy group (33.8±9.9) (p = 0.02). In fact, 90% of the PVL group and 33% of the healthy group were required to perform the VSWMT on at least a second occasion. The intra-observer reliability obtained by the Cohen’s Kappa Coefficient was 0.80, and the inter-observer reliability was 0.79.

### EEG Power Spectral Analysis

#### Group factors

**Figure 4:**
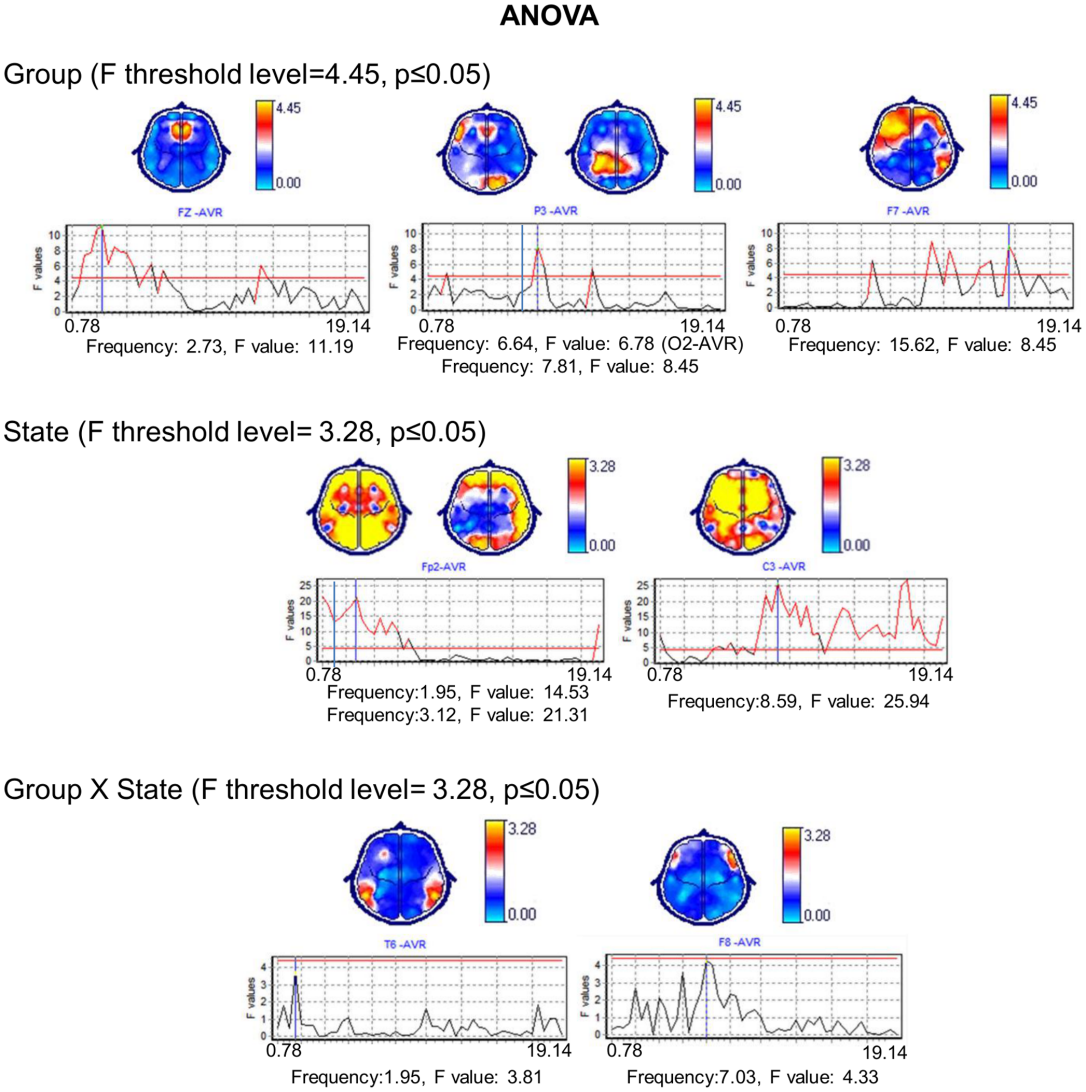
ANOVA global results. Representative frequencies are shown accompanied by a graph with all of the narrow-band frequencies included in the X-axis and the F values in the Y-axis. The represented frequencies are marked by vertical blue lines that cross the frequency in question. The threshold levels (p≤0.05) are shown by horizontal red lines. Topographic maps show in yellow the cerebral regions in which the significant differences were identified.

The global ANOVA revealed significant between-groups differences in the diverse frequencies and regions. Significant differences were identified in the frontocentral regions in frequencies from 1.56 to 5.86 Hz, in the left frontal, parietal and right occipital regions from 6.64 to 8.20 Hz and in the bilateral frontal and right temporal regions from 9.77 to 19.53 Hz (Figure 4).

### Post-hoc Analysis Between the Groups

#### Baseline

**Figure 5:**
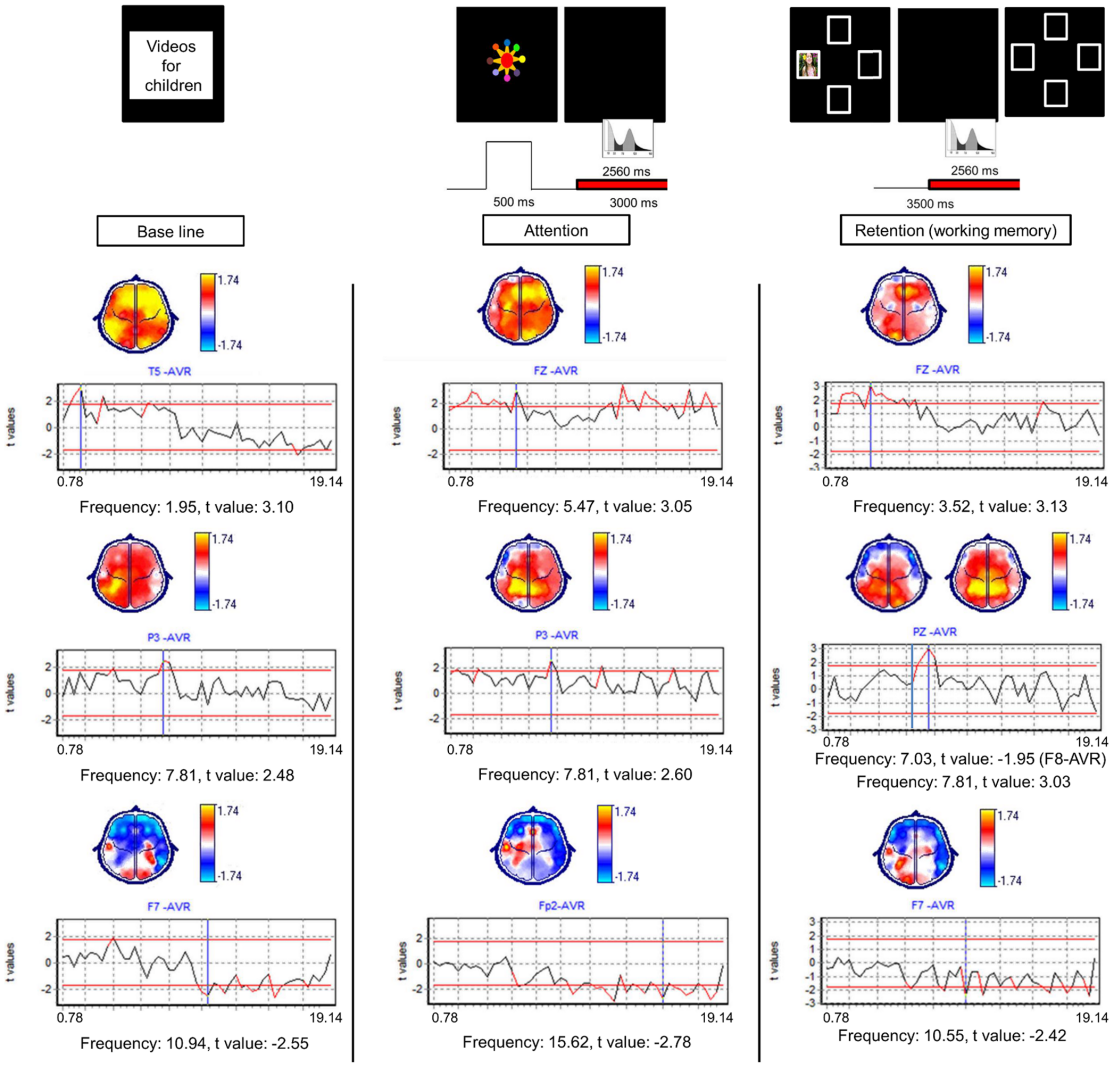
Post-hoc t-test comparing differences between groups in each of the conditions. On the Y-axis, t values are shown. Positive t values, shown in yellow on the topographic map, imply higher power in the PVL group compared to the healthy group. Negative t values, shown in blue on the topographic map, imply lower power in the PVL group compared to the healthy group.

The PVL group had higher absolute power than did the healthy group in the frontocentral, temporal, occipital and left parietal regions in frequencies from 1.17 to 8.03 Hz. In contrast, the PVL group had significantly lower absolute power than did the healthy group in the bilateral frontal and right temporal regions in frequencies from 9.77 Hz to 19.14 Hz (Figure 5).

#### Attention

The PVL group had higher absolute power than did the healthy group in the frontocentral, left parietal and bilateral frontal, central and occipital regions in frequencies from 1.17 to 5.86 Hz. The PVL group also had higher absolute power than did the healthy group in the frontocentral, bilateral parietal regions, left temporal and right occipital regions in frequencies from 6.64 to 19.14 Hz. In contrast, the PVL group had lower absolute power than did the healthy group in the bilateral frontal and right temporal regions in frequencies from 6.64 to 19.14 Hz (Figure 5).

#### WM retention

The PVL group had significantly higher absolute power than did the healthy group in the frontocentral, left temporoparietal and bilateral occipital regions in frequencies from 1.17 to 6.64 Hz and in the centroparietal and left parietal regions in frequencies from 7.03 to 8.20 Hz. As in the other conditions, the PVL group had lower absolute power than did the healthy group in the bilateral frontal and right temporal regions in frequencies from 7.03 to 19.14 Hz (Figure 5).

#### Condition factor

Significant differences were identified between the baseline, attention and WM conditions in all of the cerebral regions in frequencies from 1.17 to 7.03 Hz. In the bilateral frontocentral, left temporoparietal and right occipital regions, the frequencies ranged from 7.42 to 9.77 Hz, and in the left frontal and bilateral centroparietal regions, they ranged from 10.16 to 19.14 Hz (Figure 4).

### Post-hoc Analysis Between Conditions

#### Baseline versus attention

**Figure 6:**
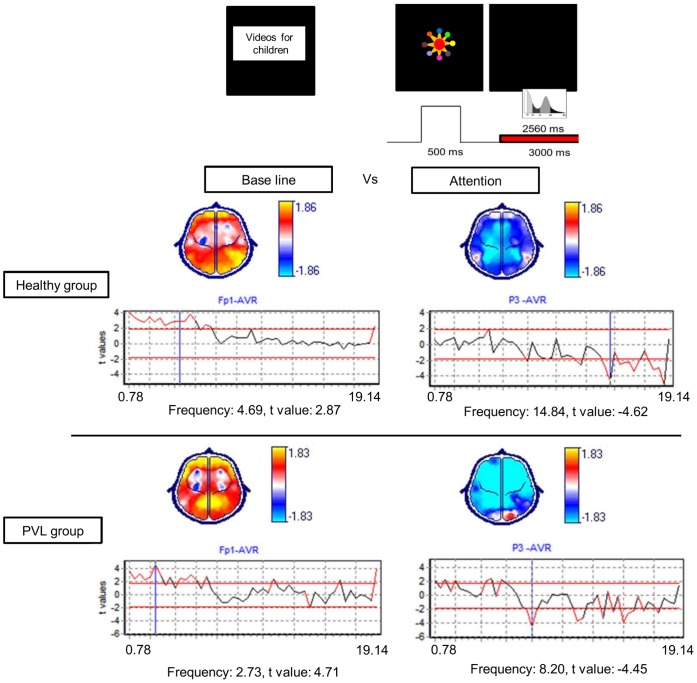
Post-hoc t-test comparing the differences between the baseline and attention conditions in each group. Positive t values, shown in yellow on the topographic map, imply power increases during the attention condition compared to baseline. Negative t values, shown in blue on the topographic map, imply power decreases during the attention condition compared to the baseline.

The healthy group showed absolute power increases in attention compared to baseline in the bilateral frontal, right temporoparietal and left occipital regions in frequencies from 1.17 to 7.03 Hz. Absolute power decreases were observed in the left frontal, central, parietal and occipital regions in frequencies from 7.81 to 19.14 Hz (Figure 6). In contrast, the PVL group had absolute power increases in attention compared to baseline in the bilateral frontal, parietal, occipital and right temporal regions in frequencies from 1.17 to 7.03 Hz. Absolute power decreases were observed in almost all of the cerebral regions except the occipital regions in frequencies from 7.42 to 19.14 Hz (Figure 6).

#### Attention versus WM

**Figure 7:**
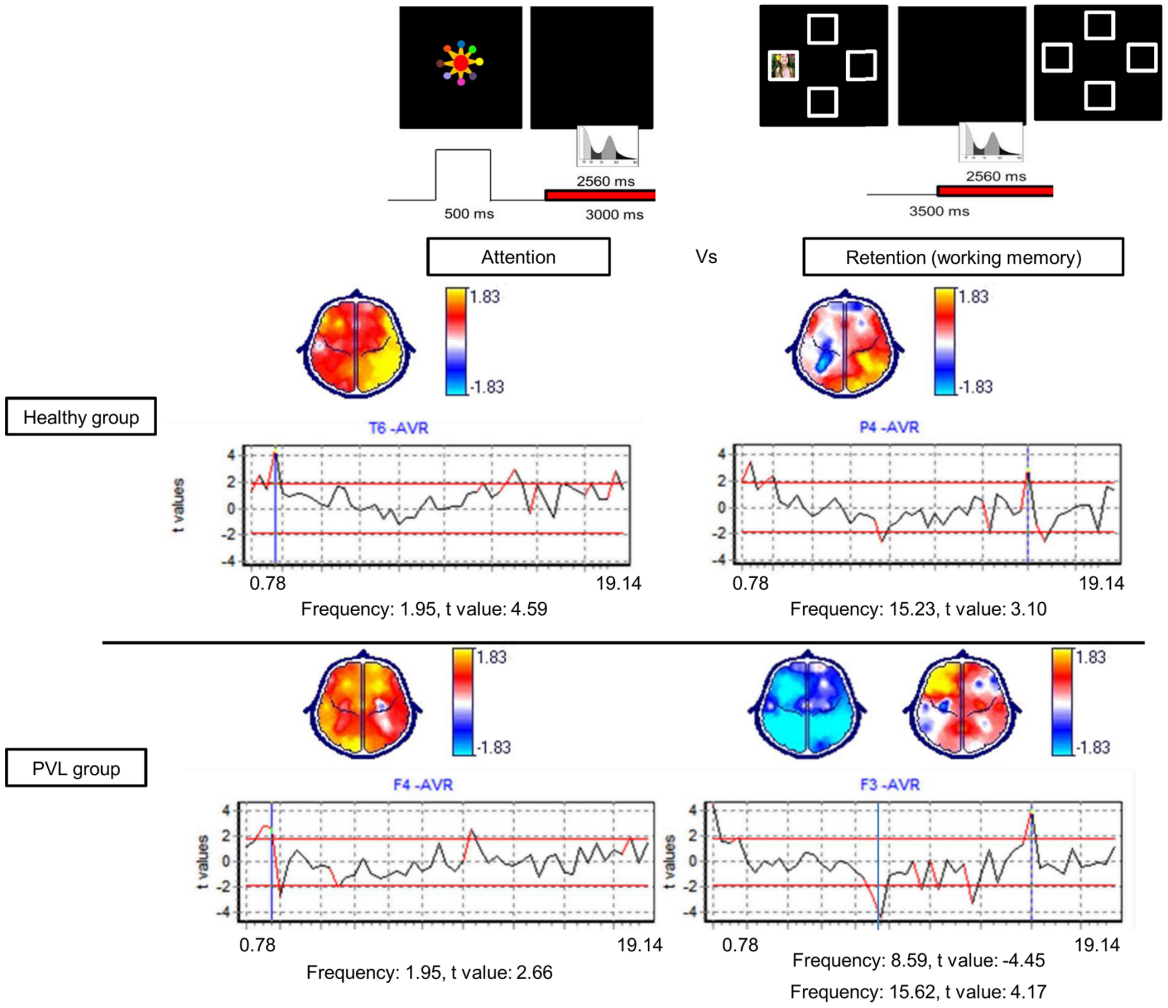
Post-hoc t-test comparing differences between the attention and WM conditions in each group. Positive t values, shown in yellow on the topographic map, imply power increases during WM in comparison with attention. Negative t values, shown in blue on the topographic map, imply power decreases during WM in comparison with attention.

The healthy group had absolute power increases in WM compared to attention in the bilateral frontal and right temporal, parietal and occipital regions in frequencies from 1.17 to 2.34 Hz, in the left temporal regions in frequencies from 3.12 to 3.52 Hz, over the right temporal regions at 5.46 Hz and in the right temporal, parietal and occipital regions in frequencies from 14.06 to 15.23 Hz (Figure 7). In contrast, the PVL group had absolute power increases during WM compared to attention in the bilateral fronto-parietal, left central and occipital regions in frequencies from 1.17 to 3.52 Hz and in the bilateral frontal and right temporal regions in frequencies from 9.37 to 19.14 Hz and absolute power decreases over the bilateral frontal, temporal, central and occipital regions in frequencies from 3.91 to 8.98 Hz (Figure 7).

### Interactions

There were significant differences between the groups and between the conditions at the following frequencies: 1.95, 7.03 and 7.42 Hz. In the WM retention condition, the PVL group showed absolute power decreases in the left temporal regions at 1.95 Hz; in contrast, the healthy group showed absolute power increases in the right temporal regions at 1.95 Hz and in the right frontal regions at 7.03 and at 7.42 Hz (Figure 4).

## Discussion

The aim of our study was to compare behavioral performance and EEG activity during a VSWMT of healthy toddlers and toddlers with a history of PVL. The main finding of our study was that the PVL group exhibited significantly worse execution in the WM task that was related to absolute power increases during WM compared to attention in the bilateral frontoparietal, left central and occipital regions in frequencies ranging from 1.17 to 3.52 Hz and in the bilateral frontal and right temporal regions in frequencies ranging from 9.37 to 19.14 Hz. Absolute power decreases were observed in the bilateral frontal, temporal, central and occipital regions in frequencies ranging from 3.91 to 8.98 Hz. In contrast, the healthy group exhibited better execution in the WM task that was related to absolute power increases during WM compared to attention in the bilateral frontal and right temporo-parieto-occipital regions at the following frequencies: 1.95, 2.34 and 15.23 Hz. These frequencies and regions have been reported as directly involved in WM processes [8], [12], [15], [16], [18], [23]. Details of these results will be described in the following paragraphs.

Regarding the clinical characteristics of the sample, it is well known that PVL presents more frequently in premature children born between 24 and 32 weeks of gestation [24] and has an incidence rate than ranges from 3 to 20% in VLBW infants who weigh between 500 and 1500 g [25] at birth. Moreover, almost 50% of premature children will develop cognitive deficits [26]. In fact, the PVL toddlers in this study had significantly lower weeks of gestation, birth weight, APGAR scores and MDIs than did the healthy toddlers, as has been reported previously [23], [24], [25].

Regarding the behavioral results, we chose a typical delayed response task with a 3.5 s delay and 4 locations. It has been reported that toddlers at 22 months are able to respond correctly with delays of 10 seconds and 8 different locations and even up to 30 s with 3 different locations [27], [28]. As shown, all of the toddlers that participated in this study were able to respond to the chosen task.

The PVL toddlers had an average of 16.1 correct responses, compared to an average of 20.5 correct responses in the healthy group. It has been described that PVL children exhibit serious motor, sensory and cognitive impairments, depending on the place and severity of the lesion [29]. In particular, visual impairments, deficits in object recognition, visual imagery and visuospatial memory have been reported [7], [30]. The results of this study indicate that, in addition to these previously reported impairments, PVL is associated with later disturbances in visuospatial WM.

With respect to the types of errors observed during the cognitive tasks, it has been proposed that infants and preschoolers born preterm have problems with the inhibition of attention to irrelevant task-features [31], [32]. This pattern suggests difficulties with the control of sustained attention [33]. The results of this study also demonstrated that the PVL toddlers presented more no-responses than did the healthy toddlers. Although this result did not reach statistical significance, it is likely that the PVL toddlers would have made more no-responses due to problems in sustained attention. In this context, it has been proposed that generalized desynchronization or power decreases from 7.02 to 10.92 Hz are related to the attentional demands of the task and sustained attention [34], [35]. In fact, in a study comparing execution and EEG changes between adults and children in the Sternberg task, the adults presented a generalized desynchronization during memory. As the children did not show any power increases in the frontal lobes at 7.8 Hz, the authors suggested that the frontal lobes of children are immature [23]. As in the last study, we identified power decreases in these frequencies during the attention and WM retention conditions in healthy subjects but observed more-pronounced and generalized power decreases in the PVL group, which may reflect sustained attention problems in the latter group.

Likewise, on average the toddlers with a history of PVL needed more trials to complete the task (44.1) than did the healthy toddlers (33.8). This finding is in line with reports that have indicated that very preterm toddlers took longer to learn and to complete the retrieval sequence of a multi-search location task [36]. As a whole, taking together the number of correct responses, no-responses and number of trials necessary to complete the task, it has been postulated that toddlers with a history of PVL exhibit visuospatial WM problems that are likely secondary to grey and white matter damage in such structures as the cortex, thalamus and cortical pathways [3], [5].

In relation to the EEG power analysis, some differences between the PVL and the healthy groups were noted in the baseline condition. The PVL group had more absolute power in frequencies ranging from 1.17 to 8.20 Hz. In contrast, the healthy group had more absolute power in higher frequencies (10.55 to 19.14 Hz) in the frontal and right temporal regions. Normal EEG neurodevelopment is characterized by slow decreases in the absolute power of low frequencies in the delta and theta ranges, accompanied by increases in higher frequencies in the alpha and beta ranges [37]. The predominance of low frequencies and lower power at high frequencies in the group of children with PVL compared with the healthy group supports the possibility that the toddlers with PVL had less-developed brain electrical oscillations. This pattern could be related to all of the reported neurological and cognitive impairments in this type of neurological damage [9]. With some variations in the frequencies or regions, this pattern of dominance of low frequencies in the toddlers with PVL was observed during the baseline, attention and WM conditions.

However, the comparison of the baseline, attention and working WM conditions indicated different electrophysiological characteristics in the two groups. For the attention condition, the healthy group exhibited absolute power increases in frequencies ranging from 1.17 to 7.03 Hz in the bilateral fronto-polar, parietal and temporal regions. In contrast, in the PVL group, absolute power increases were also noted in the same range frequencies but over almost all cerebral regions. As it is well known, such anatomical structures as the lateral geniculate nucleus, the pulvinar, the superior colliculus, the striate and extrastriate areas, the inferotemporal cortex, the superior parietal lobule and the frontal eye fields are related to the visual attention networks [38]. Thus, in the healthy group, the activation of some expected areas related to normal attention networks, such as the frontal temporal and parietal regions, were determined by absolute power increases. In contrast, the PVL group exhibited absolute power increases in the broader cerebral regions. It is proposed that the less-specialized and broader attention networks may be less effective and could be related with poorer execution in the VSWMT [7].

In the WM condition, the healthy group exhibited absolute power increases in frequencies ranging from 1.17 to 2.34 Hz in the bilateral frontal and right temporo-parieto-occipital regions and in frequencies ranging from 14.06 to 15.23 Hz in the right temporo-parieto-occipital regions. These brain areas are directly involved in the visuospatial WM circuitry [8]. In adults performing WM tasks, power increases in the theta, alpha, beta and gamma bands have been described [23], [39], [40]. In this study, the healthy toddlers presented higher absolute power at 1.95 Hz and at 15.23 Hz, which are frequencies that may resemble the theta and gamma frequencies with power increases that have been presented in adults performing WM tasks.

In contrast, the PVL group exhibited a different pattern than was observed in the healthy group during the WM condition. Absolute power increases were observed in frequencies ranging from 1.95 Hz and 14.45 to 16.41 Hz, but they were circumscribed only over the bilateral frontal and left occipital regions and not over the right posterior regions, as in the healthy subjects. PVL typically affects the periventricular white matter, involving occipital brain regions [3]. In fact, volume reductions of the temporo-parieto-occipital and frontal grey matter and the alteration of normal lateralization of white matter volumes in the parieto-occipital region (smaller right-sided structures) have been described [41]. WM impairments in the PVL group may have been secondary to diffuse damage in the posterior regions that are typically affected in PVL.

Moreover, power increases at 3.12 and 5.46 Hz were shown in the healthy group but not in the PVL group. These frequency increases have been related to the inhibition of previous information to allow for the actualization of new information in the memory storage and with the participation of the attentional processes of the central executive system, respectively [42], [23]. Even 5.46-Hz current increases have been related to memory load increases [35].

In the interactions analysis, the results indicated that the frequencies that were different between the groups and the conditions were 1.95 Hz over the right temporal regions and 7.03 and 7.42 Hz over the right frontal regions. These three frequencies were higher in the healthy group only during the WM condition. In this respect, it has been shown that during verbal recall tasks, healthy toddlers showed generalized power increases in the 3–5 Hz and the 6–9 Hz bands [16]. The results are in accordance with previous work in healthy toddlers that showed power increases in the 7.03 and 7.42 Hz bands, but not in the power increases in lower-frequency bands (1.95 Hz). This difference may reflect the fact that the authors used a broad-band power analysis during a verbal recall task [16]; we used a narrow-band power analysis during our VSWMT. We propose that this type of analysis is advantageous because it allows discriminate small changes in a broader range of frequencies.

There were some limitations of our study, one of which was the small sample sizes of the groups. In addition, the diagnosis of PVL was made with conventional MR studies. More precise techniques to diagnose PVL, such as apparent diffusion coefficient (ADC) or fractional anisotropy (FA), would have provided information about the architecture of neural pathways and would have improved the PVL diagnosis, thus allowing for a correlation of the WM impairments with the anatomic abnormalities [43]. Future studies may consider larger samples and a more complicated task with longer delay periods and more items to memorize; it would also be interesting to relate WM impairments with anatomic abnormalities.

In conclusion, toddlers with PVL exhibited lower scores on the WM task, with greater absolute power of the low-frequency range in the baseline, attention and WM conditions and with less specialized neuronal networks for WM. The group of healthy children exhibited increased absolute power in the bilateral frontal and the right temporo-parieto-occipital regions, areas that are similar to those that have been reported in infants and adults, although at lower frequencies.

## References

[1] Jensen FE (2006) Developmental factors regulating susceptibility to perinatal brain injury and seizures. Curr Opin Pediatr 18: 628–633. 17099361 10.1097/MOP.0b013e328010c536

[2] Miller SP, Shevell MI, Patenaude Y, O’Gorman AM (2000) Neuromotor spectrum of periventricular leukomalacia in children born at term. Pediatr Neurol 23: 155–159. 11020641 10.1016/s0887-8994(00)00172-7

[3] Volpe JJ (2009) Brain injury in premature infants: a complex amalgam of destructive and developmental disturbances. Lancet Neurol 8: 110–124. 19081519 10.1016/S1474-4422(08)70294-1 PMC2707149

[4] Inder TE, Huppi PS, Warfield S, Kikinis R, Zientara GP, et al (1999) Periventricular white matter injury in the premature infant is associated with a reduction in cerebral cortical gray matter volume at term. Ann Neurol 46: 755–60. 10553993 10.1002/1531-8249(199911)46:5<755::aid-ana11>3.0.co;2-0

[5] Ligam P, Haynes RI, Folkerth RD, Liu L, Yang M, et al (2009) Thalamic Damage in Periventricular Leukomalacia: Novel Pathologic Observations Relevant to Cognitive Deficits in Survivors of Prematurity. Pediatr Res 65: 524–529. 19127204 10.1203/PDR.0b013e3181998baf PMC2713790

[6] Mesulam MM (2000) Principles of Behavioral and Cognitive Neurology. New York: Oxford University Press. 540 p.

[7] Fazzi E, Bova S, Giovenzana A, Signorini S, Uggetti C, et al (2009) Cognitive visual dysfunctions in preterm children with periventricular leukomalacia. Dev Med Child Neurol 51: 974–981. 19416337 10.1111/j.1469-8749.2009.03272.x

[8] Fuster JM (2009) Cortex and Memory: Emergence of a New Paradigm. J Cogn Neurosci 21: 2047–2072. 19485699 10.1162/jocn.2009.21280

[9] Reznick JS, Morrow JD, Goldman BD, Snyder J (2004) The onset of working memory in infants. Infancy 6: 145–154.

[10] Hunter WS (1917) The delayed reaction in a child. Psychol Rev 24: 74–87.

[11] Diamond A (1985) Development of the ability to use recall to guide action, as indicated by infants performance on A not B. Child Dev. 56: 868–883. 4042750

[12] Diamond A, Doar B (1989) The performance of human infants on a measure of frontal cortex function, the delayed-response task. Dev Psychobiol 22: 271–294. 2707496 10.1002/dev.420220307

[13] Pelphrey K, Reznick SR, Goldman BD, Sasson N, Morrow J, et al (2004) Development of Visuospatial Short-Term Memory in the Second Half of the 1st Year. Dev Psychol 40: 836–851. 15355170 10.1037/0012-1649.40.5.836

[14] Napflin M, Wildi M, Sarnthein J (2008) Test-retest reliability of EEG spectra during a working memory task. Neuroimage 43: 687–693. 18817882 10.1016/j.neuroimage.2008.08.028

[15] Bell MA (2002) Power changes in infant EEG frequency bands during a spatial working memory task. Psychophysiology 39: 450–458. 12212637 10.1017/S0048577201393174

[16] Cuevas K, Raj V, Bell MA (2012) A frequency band analysis of two-year-olds’ memory processes. Int J Psychophysiol 83: 315–322. 22137966 10.1016/j.ijpsycho.2011.11.009 PMC3303934

[17] Wolfe CD, Bell MA (2004) Working memory and inhibitory control in early childhood: Contributions from electrophysiology, temperament, and language. DevelopmentalPsychobiology 44: 68–83. 10.1002/dev.10152 14704991

[18] Bell MA, Wolfe CD (2007) Changes in brain functioning from infancy to early childhood: Evidence from EEG power and coherence during working memory tasks. Dev Neuropsychol 31: 21–38. 17305436 10.1207/s15326942dn3101_2

[19] Bayley N (1993) Bayley Scales of Infant Development. San Antonio, TX: Psychological Corp.

[20] Katona F, Berényi M (2001) How early is too late? Clin Neurosci 54: 196–206.

[21] Lang PJ, Bradley MM, Cuthbert BN (2008) International affective picture system (IAPS): Affective ratings of pictures and instruction manual. Technical Report A-8. University of Florida, Gainesville, FL.

[22] Hernandez JL, Valdes P, Biscay R, Virues T, Szava S, et al (1994) A global scale factor in brain topography. Int J Neurosci 76: 267–278. 7960483 10.3109/00207459408986009

[23] Fernández T, Harmony T, Gersenowies J, Silva-Pereyra J, Fernández-Bouzas A, et al (2002) Sources of EEG activity during a verbal working memory task in adults and children. Clin Neurophysiol 2002 54: 269–283.

[24] Kinney HC, Back SA (1998) Human oligodendrocyte development: relationship to periventricular leukomalacia. Semin Pediatr Neurol 5: 180–189. 9777676 10.1016/s1071-9091(98)80033-8

[25] Inder TE, Wells SJ, Mogridge NB, Spencer C, Volpe JJ (2003) Defining the nature of the cerebral abnormalities in the premature infant: a qualitative magnetic resonance imaging study.J Pediatr. 143: 171–179. 10.1067/S0022-3476(03)00357-3 12970628

[26] Marlow N, Wolke D, Bracewell M, Samara M (2005) Neurologic and developmental disability at six years after extremely preterm birth. N Engl J Med 352: 9–19. 15635108 10.1056/NEJMoa041367

[27] Kagan J (1981) The second year: The Emergence of Self-Awareness. Cambridge, MA: Harvard University Press. 163p.

[28] Barth J, Call J (2006) Tracking the displacement of objects: A series of tasks with great apes (Pan troglodytes, Pan paniscus, Gorilla gorilla, and Pongo pygmaeus) and young children (Homo sapiens). J Exp Psychol Anim Behav Process 32: 239–252. 16834492 10.1037/0097-7403.32.3.239

[29] Blumenthal I (2004) Periventricular leucomalacia: a review. Eur J Pediatr 163: 435–442. 15179510 10.1007/s00431-004-1477-y

[30] Ricci D, Anker S, Cowan F, Pane M, Gallini F, et al (2006) Thalamic atrophy in infants with PVL and cerebral visual impairment. Early Hum Dev 82: 591–595. 16500047 10.1016/j.earlhumdev.2005.12.007

[31] Sun J (2003) Early indicators of executive function and attention in preterm and full-term infants. PhD Thesis. Brisbane, Australia: Queensland University of Technology.

[32] Espy KA, Stalets MM, McDiarmid MM, Senn TE, Cwik MF, et al (2002) Executive functions in preschool children born preterm: Application of cognitive neuroscience paradigms. Child Neuropsychol 8: 83–92. 12638062 10.1076/chin.8.2.83.8723

[33] van de Weijer-Bergsma E, Wijnroks L, Jongmans MJ (2008) Attention development in infants and preschool children born preterm: A review. Infant Behav Dev 31: 333–351. 18294695 10.1016/j.infbeh.2007.12.003

[34] Klimesch W (1999) EEG alpha and theta oscillations reflect cognitive and memory performance:a review and analysis. Brain Res. Rev 29: 169–195. 10.1016/s0165-0173(98)00056-3 10209231

[35] Gevins A, Smith M, Mc Evoy L, Yu D (1997) High-resolution EEG mapping of cortical activation related to working memory: effects of task difficulty, type of processing, and practice. Cereb Cortex 7: 374–385. 9177767 10.1093/cercor/7.4.374

[36] Woodward LJ, Edgin JO, Thomson D, Inder TE (2005) Object working memory deficits predicted by early brain injury and development in the preterm infant. Brain128: 2578–2587. 10.1093/brain/awh618 16150850

[37] Otero GA, Harmony T, Pliego-Rivero FB, Ricardo-Garcell J, Bosch-Bayard J, et al (2011) QEEG norms for the first year of life. Early Hum Dev 87: 691–703. 21696895 10.1016/j.earlhumdev.2011.05.010

[38] Kastner S, Pinsk MA (2004) Visual attention as a multilevel selection process. Cogn Affect Behav Neurosci 4: 483–500. 15849892 10.3758/cabn.4.4.483

[39] Jokisch D, Jensen O (2007) Modulation of Gamma and Alpha Activity during a Working Memory Task Engaging the Dorsal or Ventral Stream. J Neurosci 27: 3244–3255. 17376984 10.1523/JNEUROSCI.5399-06.2007 PMC6672464

[40] Lisman J, Buzsáki G (2008) A Neural Coding Scheme Formed by the Combined Function of Gamma and Theta Oscillations. Schizophr Bull 34: 974–980. 18559405 10.1093/schbul/sbn060 PMC2518638

[41] Kesler SR, Ment LR, Vohr B, Pajot SK, Schneider KC, et al (2004) Volumetric analysis of regional cerebral development in preterm children. Pediatr Neurol 31: 318–325. 15519112 10.1016/j.pediatrneurol.2004.06.008 PMC3061618

[42] Harmony T, Fernández T, Silva J, Bosch J, Valdés P, et al (1999) Do specific EEG frequencies indicate different processes during mental calculation? Neurosci. Lett 266: 25–28. 10.1016/s0304-3940(99)00244-x 10336175

[43] Beaulieu C (2002) The basis of anisotropic water diffusion in the nervous system - a technical review. NMR in Biomedicine 15: 435–455. 12489094 10.1002/nbm.782

